# Urothelial Carcinoma of the Penile Urethra as a Potential Secondary Complication of Junctional Epidermolysis Bullosa: A Case Report and Review of the Literature

**DOI:** 10.1155/crdm/5549697

**Published:** 2026-02-23

**Authors:** Jessica McClatchy, Ingrid Winship, Laura Scardamaglia, Vanessa Morgan, Gayle Ross

**Affiliations:** ^1^ Dermatology Department, The Royal Melbourne Hospital, Melbourne, Victoria, Australia, thermh.org.au; ^2^ College of Medicine and Public Health, Flinders University, Adelaide, South Australia, Australia, flinders.edu.au; ^3^ Faculty of Medicine Dentistry and Health Sciences, The University of Melbourne, Melbourne, Victoria, Australia, unimelb.edu.au; ^4^ Genetics Department, The Royal Melbourne Hospital, Melbourne, Victoria, Australia, thermh.org.au; ^5^ Dermatology Department, Western Health, Footscray, Victoria, Australia, westernhealth.org.au; ^6^ Dermatology Department, The Royal Children’s Hospital, Melbourne, Victoria, Australia, rch.org.au

## Abstract

Junctional epidermolysis bullosa is a rare autosomal recessive genetic dermatosis which is characterised by cutaneous and mucosal blistering. Cutaneous squamous cell carcinomas arising in areas of chronic wounds and scarring are a well‐recognised complication. Mucosal involvement of the respiratory, urogenital and gastrointestinal tract can occur, though reports of associated mucosal carcinomas are scarce. We present a case of an 81‐year‐old male with junctional epidermolysis bullosa, multiple metastatic cutaneous squamous cell carcinomas and a papillary urothelial carcinoma. He was diagnosed with an invasive, high‐grade pT2 papillary urothelial carcinoma of the penile urethra at age 74. This was initially identified by the patient as a nonhealing ulcer adjacent to the penile meatus on a background of recurrent blistering of the glans penis. Staging imaging revealed no nodal enlargement or distant metastasis. Management included a partial urethrotomy of the penile urethra, and he is currently in remission. We hypothesise that the urothelial carcinoma may have developed secondary to a permissive tumour microenvironment, which results from chronic inflammation and fibrosis in junctional epidermolysis bullosa.

## 1. Introduction

Junctional epidermolysis bullosa (JEB) is a rare autosomal recessive genetic dermatosis which is characterised by cutaneous and mucosal blistering. While generalised severe JEB is usually lethal within the first 12–24 months of life, the generalised intermediate JEB (JEB‐GI) subtype is less severe, and patients survive into adulthood [1]. Cutaneous squamous cell carcinomas (SCCs) arising in areas of chronic wounds and scarring are a well‐recognised complication of JEB [2]. Mucosal involvement of the respiratory, urogenital and gastrointestinal tract can also occur [3], but reports of associated mucosal carcinomas are scarce.

We present a case of an 81‐year‐old male with generalised intermediate JEB whose disease course has been complicated by a papillary urothelial carcinoma, in addition to multiple metastatic cutaneous SCCs.

## 2. Main Text

**FIGURE 1:**
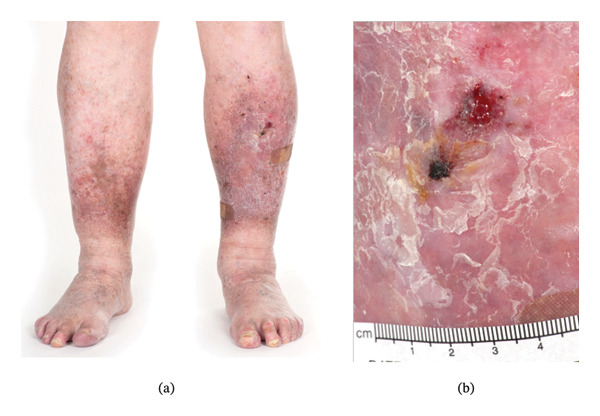
Bilateral lower legs showing lipodermatosclerosis and chronic scarring (a) from 2020. A ulcerated SCC (b) is located on the pretibial skin of the left leg and measures approximately 2.5 × 2.5 cm.

An 81‐year‐old male with JEB‐GI experienced recurrent blisters and erosions mainly affecting his limbs since childhood (Figure 1(a)). In adolescence, he was diagnosed with pemphigus vegetans and managed with high‐dose corticosteroids. His disease was refractory, and complications included Cushing’s syndrome and disseminated cryptococcus. His diagnosis was subsequently revised to epidermolysis bullosa (EB) when he was 22 years old. In 2015, at age 71, he was referred to our center for further diagnostic workup and subtyping. A diagnosis of JEB was made based on clinical features, histological examination and genetic testing. There was no family history of any skin conditions and no known consanguinity.

DNA extracted from peripheral blood revealed two variants in the *LAMB3* gene: a pathogenic heterozygous splice site variant (1132+5G > A) and a heterozygous missense variant (c.562A > G) of uncertain significance. The heterozygous missense variant NM_000228.3 (LAMB3): c.562A > G in Exon 6 is predicted to result in a minor amino acid change from lysine to glutamic acid at position 188 of the protein. Lysine at this position has low conservation (100 vertebrates, UCSC) and is located in a predicted ubiquitylation site within the laminin N‐terminal superfamily domain. In silico predictions are conflicting (PolyPhen, SIFT, CADD, MutationTaster). The variant is present in the gnomAD database at a frequency of 0.003% [4]. The variant was thus classed as a variant of uncertain significance. Coupled with the second variant which is predicted to cause a complete loss of the donor splice site (NetGene2, Fruitfly, Human Splicing Finder), we consider it may play a role in pathogenicity. The combination of these rare mutations is consistent with a diagnosis of autosomal recessive JEB.

Over the course of his life, he developed several complications of JEB including dystrophic nails, alopecia, early dental disease secondary to enamel dysplasia and multiple cutaneous SCCs. Other past medical history includes hypothyroidism, rheumatoid arthritis managed with hydroxychloroquine, ischemic cardiomyopathy and chronic lower leg venous insufficiency leading to lipodermatosclerosis.

He reported a history of multiple BCCs affecting the head and neck area from age 40. He also had a history of a scalp lentigo maligna in 2004 which was completely excised. Additionally, he was diagnosed with a keratoacanthoma affecting his right leg in 2007. His first SCC was diagnosed in 2012 at age 68, on his left pretibial lower leg.

His first metastatic SCC was identified on his right leg in 2015 when he was 71 years old, after a new tender hyperkeratotic nodule was noted on full skin examination with palpable inguinal lymphadenopathy. Histopathology revealed features consistent with a well‐differentiated SCC that was clear of margins, and 3 out of 18 lymph nodes were involved. Management consisted of a wide local excision of the primary tumor and complete inguinal lymph node dissection.

In 2019, at age 75, a further metastatic SCC of the contralateral left leg was detected on routine skin examination (Figure 1(b)). A staging positron emission tomography (PET) showed moderate uptake in the skin of the anterior left leg and marked uptake in an enlarged groin node. A wide local excision and complete left groin dissection were performed. Histopathology of the skin revealed a well‐differentiated SCC clear of margins without any perineural or lymphovascular invasion. One of the 25 nodes was positive for metastasis. Nodal metastases were thought to be secondary to the new pretibial SCC; however, recurrence from 2012 was an alternative possibility. Adjuvant radiotherapy was considered, but he was deemed a poor candidate due to concerns about skin integrity with delayed surgical wound healing and postoperative lymphoedema with his underlying JEB.

In addition to these, the patient has also had two further cutaneous SCCs limited to the skin. This included a well‐differentiated SCC of the right leg diagnosed in 2017 and a well‐differentiated SCC of the left leg diagnosed in February 2025. Both were managed with complete surgical excision.

**FIGURE 2:**
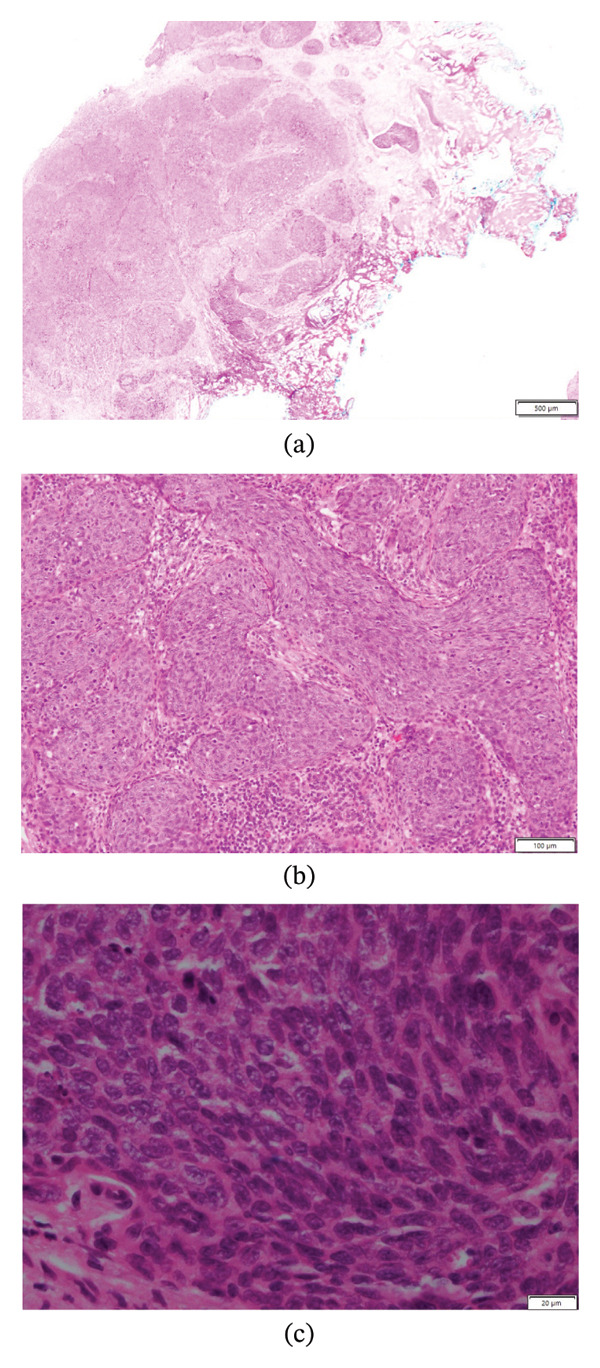
Microscopic examination of the patient’s urethral biopsy at low (a), medium (b) and high (c) power with standard haematoxylin and eosin. Solid nests of malignant epithelial cells invade into the lamina propria and focally into smooth muscle. Findings are consistent with a diagnosis of an invasive (pT2) high‐grade urothelial carcinoma.

At age 74, he was diagnosed with an invasive, high‐grade pT2 papillary urothelial carcinoma of the penile urethra. This was identified by the patient as a nonhealing area of ulceration adjacent to the penile meatus on a background of recurrent blistering of the glans penis. Biopsy confirmed the diagnosis (Figure 2), and a staging CT IVP and PET revealed no nodal enlargement or distant metastasis. This was managed surgically with a partial urethrotomy of the penile urethra. He continues to have 6‐monthly surveillance flexible cystoscopies.

He continues to experience recurrent blisters and erosions of his lower legs. These are managed supportively with dressings. He is currently in remission from the SCCs and urothelial carcinoma. He continues regular follow‐up with urology, dermatology and the EB MDT. He lives independently and reports having a good quality of life.

## 3. Discussion

To our knowledge, this case represents one of the oldest reported cases of JEB‐GI. Only one other study reports a patient of comparable age [5]. As such, this case demonstrates the natural history of JEB‐GI with complications including multiple aggressive cutaneous SCCs. Additionally, the occurrence of papillary urothelial carcinoma in our case is noteworthy. Involvement of the transitional cell epithelium of the urinary tract is known to occur in patients with JEB [3], and the development of carcinoma in our patient could possibly be related to chronic urinary tract injury and inflammation.

EB‐related SCCs (EB‐SCCs) are distinct from conventional SCCs related to ultraviolet (UV) exposure and immunosuppression. Typically, EB‐SCCs arise on the lower legs in areas of chronic wounds and scarring [2]. Similar to SCCs arising in burns, EB‐SCCs tend to be aggressive with a high propensity for metastasis and/or local relapse irrespective of their histopathological differentiation [2]. The occurrence of EB‐SCCs is most common in recessive dystrophic EB; however, patients with JEB‐GI also have an increased risk of SCCs from their third decade of life [6]. The SCCs in our patient shared many characteristics of this, including metastasising despite being well‐differentiated without lymph vascular or perineural invasion on histology. However, our patient had many risk factors of UV‐SCCs including Fitzpatrick skin phototype 2, significant cumulative sun exposure growing up in rural Queensland and a history of other skin cancers including multiple BCCs and a lentigo maligna. Thus, the origin of SCCs may be multifactorial. Given the presence of these risk factors, it is interesting that his first SCC did not occur until the patient was in his 6^th^ decade of life.

Multiple theories have been proposed to explain the occurrence of SCC in EB. Chronic wounds induced by repeated mechanical trauma, together with aberrant activation of inflammatory pathways and dermal fibrosis, establish permissive tumor microenvironments. Proinflammatory states cause fibroblasts to remain as myofibroblasts which drives the formation of a fibrotic stroma. In this environment, fibroblasts resemble carcinoma‐associated fibroblasts that promote oncogenesis through the production of cytokines, chemokines and extracellular matrix proteins that sustain tumor cell growth and migration [7]. Furthermore, the mutation burden in EB‐SCCs is high, with similarities to the genetic landscape of UV‐induced SCC including mutations in *TP53, NOTCH1, NOTCH2* and *HRAS* [7]. This has been shown to be contributed to by dysregulated activity of the APOBEC (apolipoprotein B mRNA editing enzyme catalytic polypeptide‐like) family of enzymes in recessive dystrophic EB [8]. Finally, disease‐defining mutations to laminin‐332 and collagen XVII could be implicated in tumor pathogenesis. Reduced levels of laminin‐332 are associated with increased keratinocyte migration and integrin‐mediated signaling in EB [8]. However, conflicting data exist, and the precise role of laminin 332 and collagen XVII in JEB‐SCCs needs to be fully elucidated [7].

Chronic inflammation and scarring of the urogenital tract in JEB can occur secondary to trauma and friction. Thus, it is possible that the urothelial carcinoma seen in our patient emerged secondary to an oncogenic environment by the mechanisms proposed above. Urological complications are reported in up to 28% of generalised intermediate JEB cases and include acute painful blisters and chronic scarring and fibrosis leading to stricture formation, urinary retention, bladder hypertrophy and hydronephrosis [9]. Meatal stenosis has been reported in 2.6% of JEB‐GI patients [9], and more extensive scarring has been observed in patients with laminin 332 disease compared to collagen XVII [3]. One other case of urogenital carcinoma has been reported in the literature in a patient with JEB, a case of transitional cell carcinoma of the bladder [10]. A further case of squamous metaplasia of the bladder has also been reported, which could represent a precursor to malignant lesions [11]. However, given the advanced age and male gender of this patient, it is also possible that the disease emerged independently of JEB.

## 4. Conclusion

We present a case of JEB occurring in an 81‐year‐old with concurrent urothelial carcinoma. We hypothesise that the urothelial carcinoma may have developed secondary to a fibrotic and inflamed microenvironment that promotes tumorigenesis, resulting from prolonged mucosal fragility in JEB.

## Funding

This research did not receive any funding from the public, commercial, or not‐for‐profit sectors. Open access publishing was facilitated by The University of Melbourne, as part of the Wiley ‐ The University of Melbourne agreement via the Council of Australasian University Librarians.

## Consent

Informed patient consent was obtained from the patient, including for the use of photographs.

## Conflicts of Interest

The authors declare no conflicts of interest.

## Data Availability

Data sharing is not applicable to this article as no datasets were generated or analysed during the current study.
